# MCHP (Monte Carlo + Human Phantom): Platform to facilitate teaching nuclear radiation physics

**DOI:** 10.1371/journal.pone.0257638

**Published:** 2021-09-17

**Authors:** Mehrdad Shahmohammadi Beni, Hiroshi Watabe, Dragana Krstic, Dragoslav Nikezic, Kwan Ngok Yu

**Affiliations:** 1 Department of Physics, City University of Hong Kong, Hong Kong, China; 2 Division of Radiation Protection and Safety Control, Cyclotron and Radioisotope Center, Tohoku University, Sendai, Japan; 3 Faculty of Science, University of Kragujevac, Kragujevac, Serbia; 4 State University of Novi Pazar, Novi Pazar, Serbia; Mohanlal Sukhadia University, INDIA

## Abstract

Some concepts in nuclear radiation physics are abstract and intellectually demanding. In the present paper, an “MCHP platform” (MCHP was an acronym for Monte Carlo simulations + Human Phantoms) was proposed to provide assistance to the students through visualization. The platform involved Monte Carlo simulations of interactions between ionizing radiations and the Oak Ridge National Laboratory (ORNL) adult male human phantom. As an example to demonstrate the benefits of the proposed MCHP platform, the present paper investigated the variation of the absorbed photon dose per photon from a ^137^Cs source in three selected organs, namely, brain, spine and thyroid of an adult male for concrete and lead shields with varying thicknesses. The results were interesting but not readily comprehensible without direct visualization. Graphical visualization snapshots as well as video clips of real time interactions between the photons and the human phantom were presented for the involved cases, and the results were explained with the help of such snapshots and video clips. It is envisaged that, if the platform is found useful and effective by the readers, the readers can also propose examples to be gradually added onto this platform in future, with the ultimate goal of enhancing students’ understanding and learning the concepts in an undergraduate nuclear radiation physics course or a related course.

## Introduction

**Fig 1:**
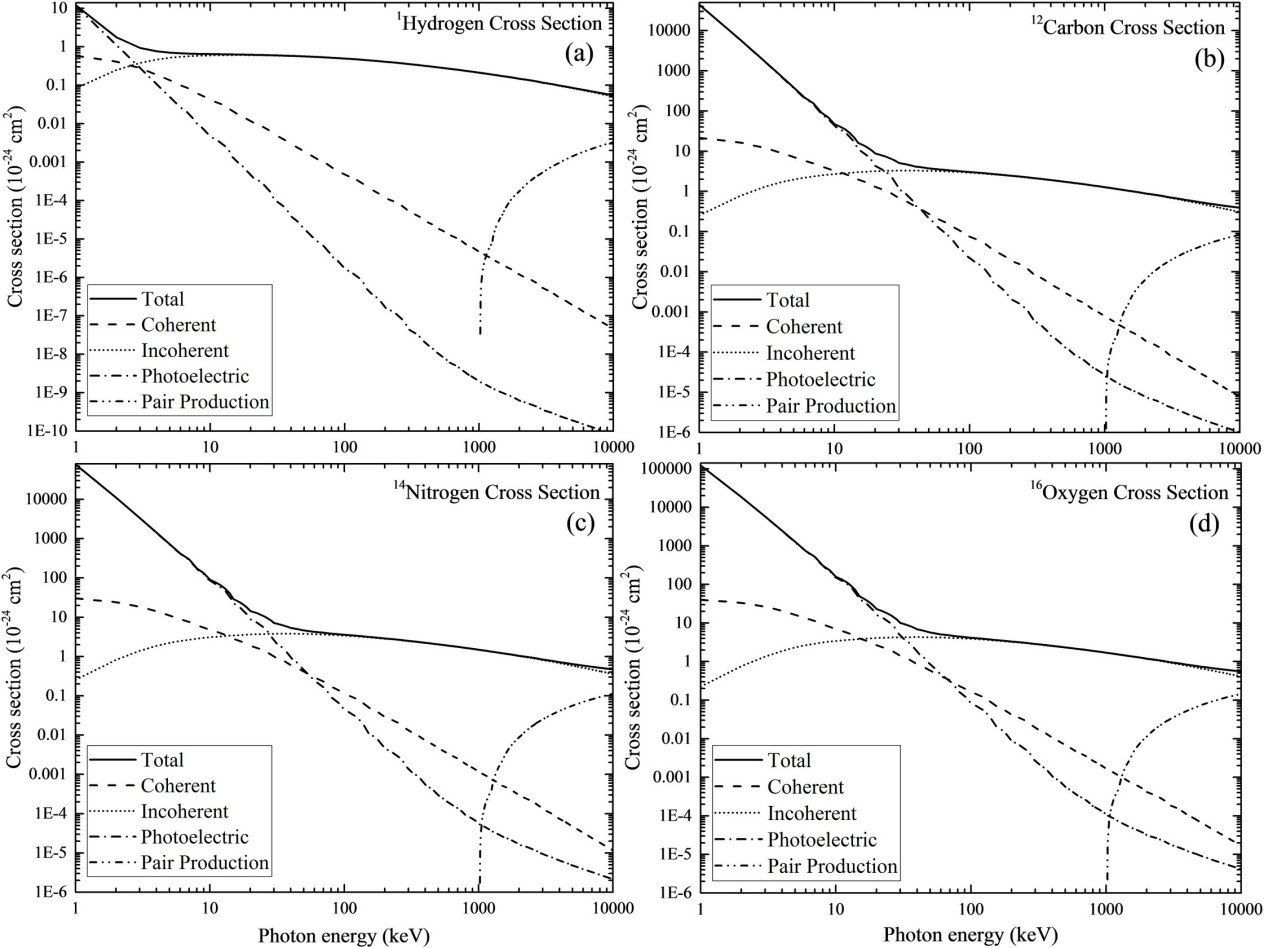
Interaction cross-section data for ^1^H, ^12^C, ^14^N and ^16^O that are the major nuclei in a modeled human cell or organ. The four different interactions shown in the figure include coherent, incoherent, photoelectric effect and pair production. These were obtained from the National Institute of Standards and Technology (NIST) XCOM library homepage: https://physics.nist.gov/PhysRefData/Xcom/html/xcom1.html.

Nuclear radiation physics is an interesting subject in physics but some concepts are abstract and intellectually demanding. The main topics in nuclear radiation physics include the nature of ionizing radiations, their interactions and detection. Notably, the stochastic nature of radioactivity as well as interactions between ionizing radiation with matter have made their direct visualization relatively tedious and challenging, and as a result many concepts involved in nuclear radiation physics are being taught nowadays using average properties and/or probabilities. For example, Fig 1 shows the photon interaction cross-section data for some elements ^1^H, ^12^C, ^14^N and ^16^O that are major nuclei in a modeled human cell or organ. These were obtained from the National Institute of Standards and Technology (NIST) XCOM library homepage: https://physics.nist.gov/PhysRefData/Xcom/html/xcom1.html. The four different interactions shown in the figure include coherent, incoherent, photoelectric effect and pair production. In another example, in the discussion of the linear attenuation coefficient *μ* of photons in a target material, which is defined as the product *n* × *σ*, where *n* is the number density of atomic nuclei in the target material (i.e., number of atomic nuclei per unit volume), while *σ* is the interaction cross section between the photons and the atomic nuclei. Such treatments of the topics might be the most convenient ones for teaching, but the general lack of visualization has inevitably made the concepts abstract, which might not have helped the students’ understanding and learning.

**Fig 2:**
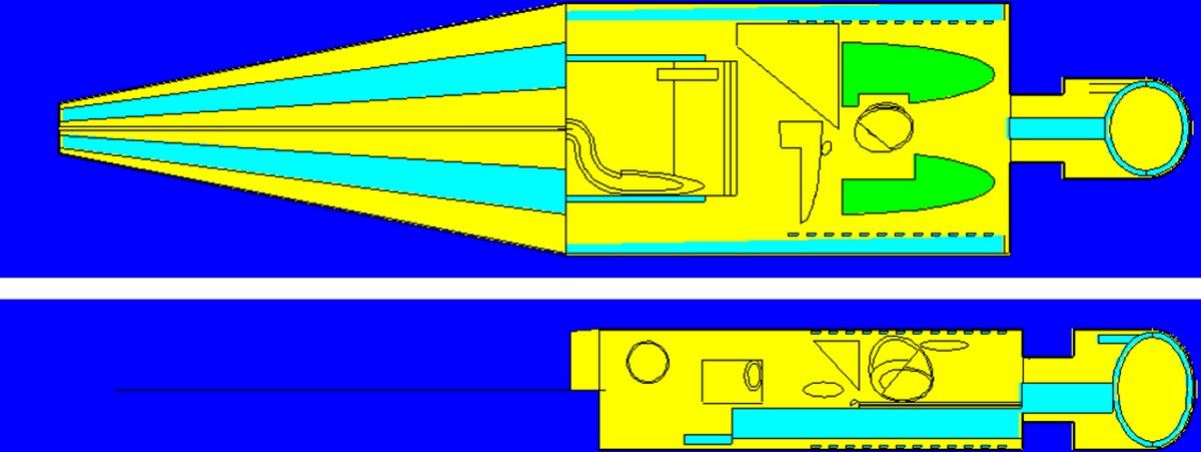
The Oak Ridge National Laboratory (ORNL) adult male human phantom [1] used in the present work.

In the present paper, we proposed an “MCHP platform” (MCHP is acronym for Monte Carlo simulations + Human Phantoms) for teaching such abstract concepts in an undergraduate nuclear radiation physics course (or a related course) through general provision of visualization. To further arouse the students’ interest, the platform involved the Oak Ridge National Laboratory (ORNL) adult male human phantom that we have previously developed [1] as shown in Fig 2, and the topics could be discussed in terms of, for examples, the radiation dose delivered to different organs in the human phantom in real time. In this way, the students could likely perceive that the discussions had a direct relevance to themselves. As mentioned above, interactions between ionizing radiation with matter are stochastic, so Monte Carlo simulations were employed to achieve direct visualization.

An example was also provided in this preliminary work to help teach the concepts of interactions between photons and matter, attenuation coefficient and radiation shielding against photons [2]. The materials and methods employed in working out this example would be described in section 2 below, while the results and discussion will be presented in section 3 below. It is envisaged that, if the platform is found useful and effective by the readers, the readers can also propose examples to be gradually added onto this platform in future, with the ultimate goal of enhancing students’ understanding and learning the concepts in an undergraduate nuclear radiation physics course or a related course.

## Methodology and numerical results

We refer interested readers to the Refs. [1–4] regarding the modelling and irradiation of the ORNL adult male human phantom. In the present example, the Monte Carlo N-Particle (MCNP) code version 5 was used to simulate the transport and interaction of high energy photons. Similar to our previous work [1], an isotropic point-like ^137^Cs radioactive source was used, which emitted photons with energy of 661.6 keV in all directions. The source to phantom distance was kept fixed at 100 cm from the face of phantom (please refer to Ref. [2] for the detailed setup). The two shielding materials considered in the present example were (1) concrete and (2) lead, both with thicknesses of 10 and 20 cm [5]. Three main organs, namely, brain, spine and thyroid were used to demonstrate the impact of the ionizing radiation on the human body. The dose from the photons in each of these three selected organs were determined.

**Fig 3:**
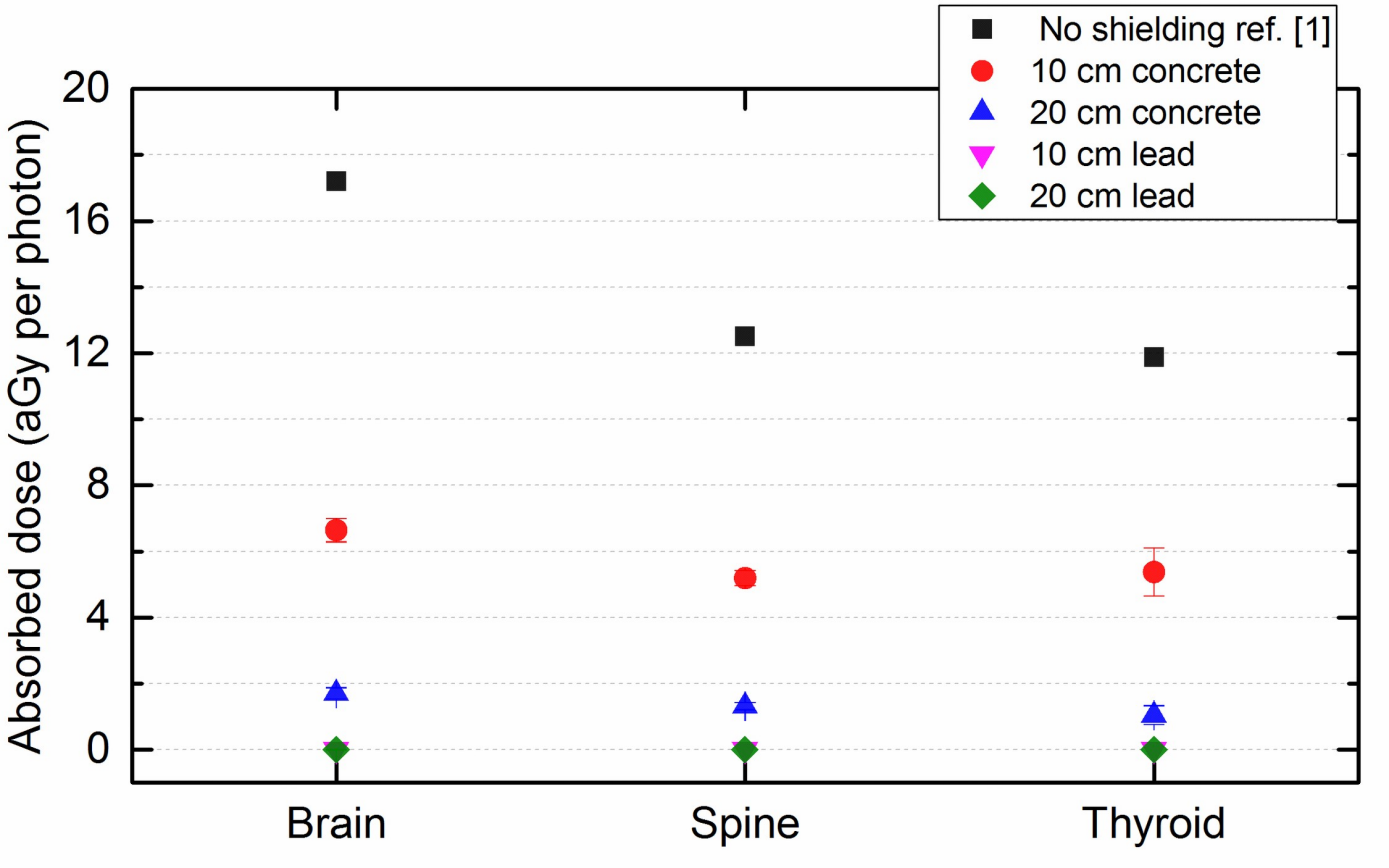
Absorbed photon dose per photon in the brain, spine and thyroid of the adult male phantom for no shielding, 10 and 20 cm thick concrete and lead shielding. The error bars represent the statistical uncertainties associated with Monte Carlo simulation. Some of the error bars are smaller than symbols.

**Fig 4:**
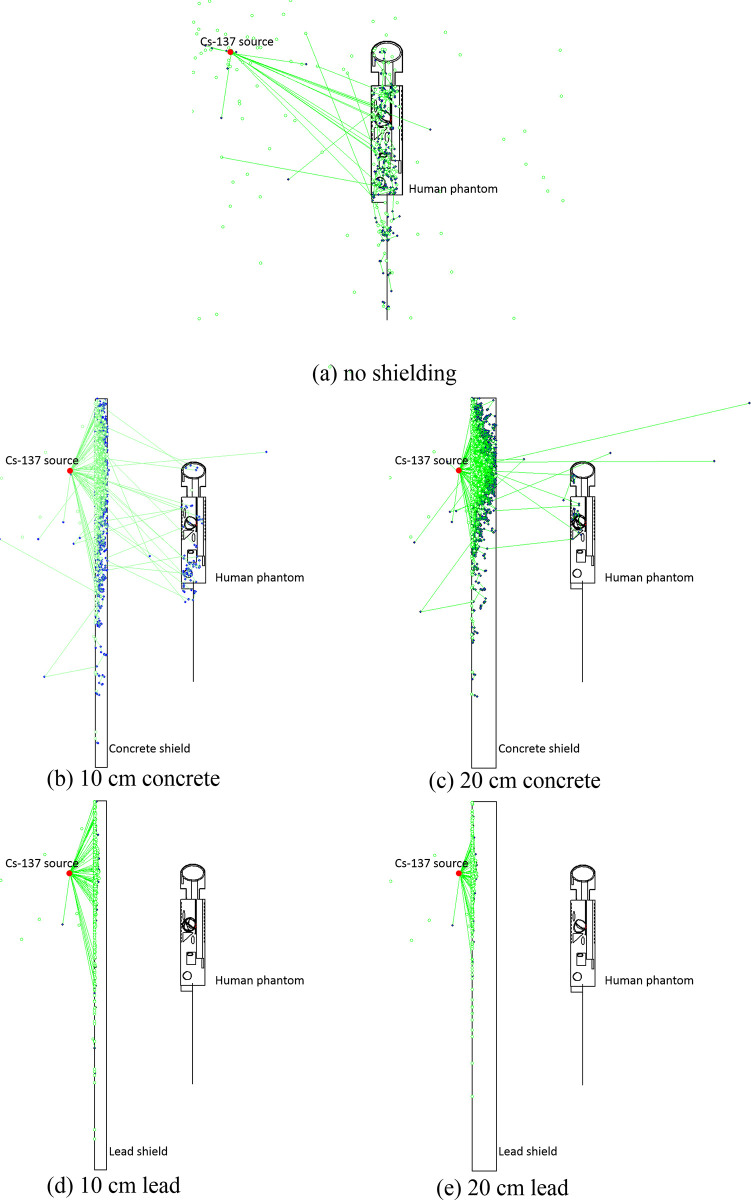
Graphical representation of photon tracks for the cases with (a) no shielding, (b) 10 cm concrete, (c) 20 cm concrete, (d) 10 cm lead and (e) 20 cm lead shielding. Blue dots: positions of photon interactions; green lines: links between positions of multiple interaction (i.e., blue dots); green open circle: positions at which photons would not undergo further multiple scattering. Some symbols may have overlapped with one another. For a better understanding, the readers could cross check with the video clips, where the symbols are displayed in sequence.

**Fig 5:**
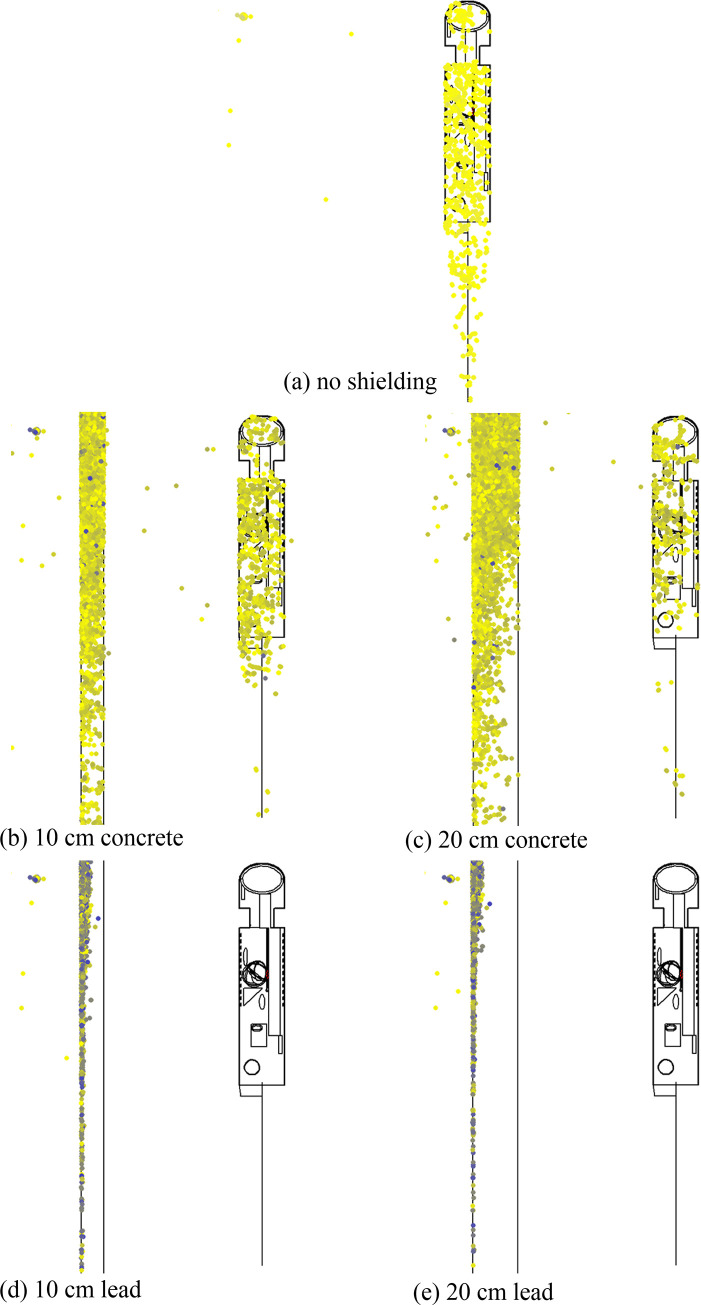
Graphical representation of the energy of interacting photons for the cases with (a) no shielding, (b) 10 cm concrete, (c) 20 cm concrete, (d) 10 cm lead and (e) 20 cm lead shielding. The lighter yellow dots represent photons with higher energy, while darker yellow dots represent photons with lower energy.

Fig 3 shows the absorbed photon dose per photon in the brain, spine and thyroid of the adult male phantom for no shielding, 10 and 20 cm thick concrete and lead shielding. Interestingly, Fig 3 shows that the absorbed photon doses per photon in the organs would be reduced when a shielding material was inserted between the source and the phantom, and the absorbed photon doses per photon would be reduced more for a thicker shield. Furthermore, the lead shields were more effective than concrete shields. The energy of the employed ^137^Cs source is 661.6 keV and at this energy the mass attenuation coefficient for concrete and lead would be about 8.236×10^−2^ and 1.248×10^−1^ cm^2^/g, respectively. In addition, the density of concrete and lead would be 2.35 and 11.35 g/cm^3^, respectively, which leads to much larger linear attenuation coefficient (mass attenuation coefficient × density) for lead than concrete for shielding against gamma rays. This can be noticed from the obtained results. Although such results would usually deem to be “expected”, the complete explanation might be more tedious. For example, it would not be trivial whether the reduction in the absorbed doses were due to the absorption of the photons due to photoelectric effect (and pair production as well for general consideration; pair production was not involved in the current example involving the photons from ^137^Cs) within the shielding material, or due to the absorption of photon energy due to Compton scattering within the shielding material. Furthermore, while photoelectric effect (and pair production for general consideration) will attenuate the number of photons reaching the human phantom (which could also be observed from the visualization in Fig 4 below) and will thus attenuate the absorbed dose in the human phantom, Compton scattering will reduce the energy of the photons reaching the human phantom (which could also be observed from the visualization in Fig 5 below) and whether that will attenuate the absorbed dose in the human phantom will further depend on the interaction cross section of the photons with the reduced energies [6, 7].

For the energy range of photons considered in the current example (where the original source was ^137^Cs), incoherent (Compton) scattering is a main photon-matter interaction mechanism [8–15] (see the cross-section data shown in Fig 2), and the dose will be delivered to the organs in the phantom mainly due to photoelectric effect and incoherent scattering. Unfortunately, the numerical absorbed photon doses alone could not help the students’ understanding and learning on photon scattering and spreading, which could however be achieved with the help of visualization in Figs 4 and 5 below.

To sum up, the results in Fig 3 were interesting, but it might not be straightforward to understand these results without direct visualization. In section 3 below, as a demonstration of the benefits from the direct visualization capability proposed in the current MCHP platform, graphical visualization snapshots as well as video clips of real time interactions between the photons and the human phantom were presented for the involved cases, and the results were explained with the help of such snapshots and video clips.

## Visualization and discussion

To illustrate the added values of the currently proposed MCHP platform, two graphical visualization snapshots for the five involved cases are shown in Figs 4 and 5, namely, (a) no shielding, (b) 10 cm concrete, (c) 20 cm concrete, (d) 10 cm lead and (e) 20 cm lead shielding (distance between the shield and the phantom was ~60 cm). The visualizations were prepared using Vised [16] that came in bundle with the MCNP package. In addition, video clips on interactions between the photons and the phantom were also prepared, corresponding to the cases with no shielding (Fig 4A), 10 cm thick concrete shield (Fig 4B) and 10 cm thick lead shield (Fig 4D). Interested readers can download these video clips from https://figshare.com/articles/media/MCHP_Monte_Carlo_Human_Phantom_Platform_to_facilitate_teaching_nuclear_radiation_physics/13798268. In Fig 4 and the video clips mentioned above, the blue dots represented the positions of photon interactions, and the green lines connected the positions of multiple interaction (i.e., blue dots). Those positions at which the photons would not undergo further multiple scattering were covered with a green open circle. It is remarked here that some symbols in Fig 4 overlapped with one another because of the high densities of dots, lines and open circles. For a better understanding, the readers could cross check with the video clips, where the symbols were shown in sequence.

Fig 4(A) is for the case with no shielding. Fig 4(B) and 4(C) represent the cases for 10 and 20 cm thick concrete shields, respectively, and the number of radiation interactions with human phantom has been significantly reduced. Fig 4(D) and 4(E) represent the cases for 10 and 20 cm thick lead shields, respectively, and almost no photons reached the human phantom. These visualizations clearly demonstrated that absorption of photons due to photoelectric effect within the shielding material contributed to the reduction in the absorbed doses in the human phantom.

On the other hand, Fig 5 shows the energy of interacting photons, using dots with different colors to represent photons with different energies. Fig 5(A) is for the case with no shielding, and the energies of the photons which interacted with the human phantom were uniform within the phantom and relatively high (as shown by lighter yellow color dots) when compared to Fig 5(B) and 5(C). Fig 5(B) and 5(C) represent the cases for 10 and 20 cm thick concrete shields, respectively, and the energies of a substantial portion of the photons which interacted with the human phantom became lower (as shown by darker yellow color dots) after having interacted with the shielding material. Fig 5(D) and 5(E) represent the cases for 10 and 20 cm thick lead shields, respectively, which show further reduction in the number and energy of the photons which interacted with the human phantom. These visualizations clearly demonstrated that the absorption of photon energy due to Compton scattering within the shielding material also contributed to the reduction in the absorbed doses in the human phantom.

The results in Figs 4 and 5 clearly revealed that the reduction in the absorbed doses per photon provided by the shielding materials were due to both absorption of the photons due to photoelectric effect, and absorption of photon energy due to Compton scattering within the shielding material. Without these visualizations, it would not be trivial to make such conclusions. As described above, the reduction in the absorbed doses were traditionally “explained” by the mass attenuation coefficient or the linear attenuation coefficient (mass attenuation coefficient × density), but this explanation did not answer the question whether the reduction in the absorbed doses were due to the absorption of the photons or due to absorption of photon energy within the shielding material. In particular, the radiation spreading of photons from the ^137^Cs source and their interactions with the phantom would enhance the understanding of students on the importance of incoherent scattering, which was a dominating photon-matter interaction mechanism over a large energy range for most commonly encountered nuclei present in the human body and other living organisms.

The video clips provided a much clearer visualization of the radiation interactions with the phantom, the stochastic nature of these interactions and the spreading of photons. In addition, it is now directly observable that after incoherent scattering of photons with the surrounding air or the shielding material, the photons would no longer be mono-energetic. Without the visualization aids, the energy distribution could only be plotted for one domain at one time. In contrast, with the help of direct visualization, changes in the photon energy in multiple domains could be examined at the same time, such as surrounding air, shielding material and human phantom (e.g., see Fig 5B and 5C).

From the above discussion, we genuinely felt that the MCHP platform proposed in the current paper could help generate “case studies” which could help undergraduate student learn the subject of nuclear radiation physics. The case studies could help explain concepts which were relatively difficult to understand using mathematical equations alone, particularly those which only described average properties, and could arouse the students’ interest since the students could easily perceive that the discussions had a direct relevance to themselves. If this MCHP platform is welcome by the community teaching nuclear radiation physics and if the platform can become popular in future, a website will be created to host the created case studies, probably with the discussions as well, which can be used by members of the teaching community. We envisage that the development of the MCHP platform can benefit both the teachers and students in a nuclear radiation physics course or a related course.

## Conclusions

The present work introduced a novel teaching platform named MCHP (acronym for Monte Carlo + Human Phantoms). Through visualization, this platform helped students better understand interactions between nuclear radiation and the human body. In the present work, irradiation by a ^137^Cs point source through different shielding materials with different thicknesses was used to demonstrate the use of the MCHP platform. Our previously developed ORNL human phantom model was employed. The MCHP platform would be very useful for pedagogical purposes. In future, we would incorporate other well-known examples in the field of nuclear radiation physics into the platform.
